# Relationship Among Traditional Semen Parameters, Sperm DNA Fragmentation, and Unexplained Recurrent Miscarriage: A Systematic Review and Meta-Analysis

**DOI:** 10.3389/fendo.2021.802632

**Published:** 2022-01-04

**Authors:** Yanpeng Dai, Junjie Liu, Enwu Yuan, Yushan Li, Ying Shi, Linlin Zhang

**Affiliations:** ^1^ Department of Clinical Laboratory, The Third Affiliated Hospital of Zhengzhou University, Zhengzhou, China; ^2^ Henan Human Sperm Bank, The Third Affiliated Hospital of Zhengzhou University, Zhengzhou, China

**Keywords:** DNA fragmentation, semen quality, recurrent miscarriage, sperm, meta-analysis

## Abstract

Several studies have explored the relationship among traditional semen parameters, sperm DNA fragmentation (SDF), and unexplained recurrent miscarriage (RM); however, the findings remain controversial. Hence, we conducted a meta-analysis to explore the relationship among traditional semen parameters, SDF, and unexplained RM. Multiple databases, including PubMed, Google Scholar, MEDLINE, Embase, Cochrane Library, Web of Science, and China National Knowledge Infrastructure (CNKI), were searched to identify relevant publications. From the eligible publications, data were extracted independently by two researchers. A total of 280 publications were identified using the search strategy. According to the inclusion/exclusion criteria, 19 publications were eligible. A total of 1182 couples with unexplained RM and 1231 couples without RM were included in this meta-analysis to assess the relationship among traditional semen parameters, SDF, and unexplained RM. Our results showed that couples with unexplained RM had significantly increased levels of SDF and significantly decreased levels of total motility and progressive motility compared with couples without RM, although significant differences were not observed in the semen volume, sperm concentration, and total sperm count between couples with and without RM. The SDF assay may be considered for inclusion in evaluations of couples with unexplained RM.

## Introduction

A uniform definition of recurrent miscarriage (RM) has not been established. The American Society for Reproductive Medicine (ASRM) defines RM as two or more consecutive miscarriages (1), while the Royal College of Obstetricians and Gynecologists (RCOG), the Chinese Society of Obstetrics and Gynecology, and the European Society of Human Reproduction and Embryology (ESHRE) guidelines define RM as three or more consecutive miscarriages (2–4). RM affects approximately 1% of couples trying to conceive (5). In almost half of the cases of RM, the etiology of the affected couples remains unclear (1). Research has mainly focused on female factors for RM, but the role of male factors in RM has recently gained attention (6–8).

Male fertility is usually assessed by the semen volume, sperm concentration, total sperm count, progressive motility, and total motility according to WHO guidelines. However, traditional semen parameters have relatively poor predictive value for spermatozoa fertilizing capacity and reproductive outcomes (9). The integrity of sperm DNA is essential for the accurate transmission of genetic information from father to offspring. Sperm DNA fragmentation (SDF) is used to assess the integrity of sperm chromatin and has been increasingly recognized as crucial because of its diagnostic potential in terms of male fertility and pregnancy outcomes. There are three main hypotheses regarding the molecular mechanism of sperm DNA damage, including oxidative stress: chromatin packaging abnormalities, and apoptosis (10). A certain degree of sperm DNA damage can be repaired by the oocyte; however, when the damage exceeds the repair capacity of the oocyte, then adverse pregnancy outcomes may occur (11). Many clinical studies have investigated the relationship between SDF and reproductive outcomes, and several studies have suggested that SDF is associated with poor fertilization, suboptimal embryo quality, and lower pregnancy rates (12–15). Gandini et al. suggest that sperm with DNA damage are capable of fertilizing an oocyte (16). However, other studies have indicated that SDF is not associated with the fertilization rate or pregnancy outcome (17, 18). Thus, the implications of SDF on fertilization rate and pregnancy outcome remain controversial.

Many SDF assays have been developed, and the main methods are as follows: sperm chromatin dispersion (SCD) (19–23), terminal deoxyuridine nick end labeling (TUNEL) (24–28), acridine orange test (AOT) (29), sperm chromatin structure assay (SCSA) (27, 30–35), and aniline blue (AB) staining (36). TUNEL is a direct method of measuring single and double DNA strand breaks by using probes, while SCD, SCSA, AOT, and AB staining are indirect methods that use the increased susceptibility of sperm DNA damage to acid-induced denaturation.

This systematic review and meta-analysis aimed to assess the relationship among traditional semen parameters, SDF, and unexplained RM.

## Materials and Methods

### Literature Search

The study was performed in accordance with the Preferred Reporting Items for Systematic Reviews and Meta-Analyses (PRISMA) guidelines (37). Multiple databases, including PubMed, Google Scholar, Cochrane Library, Embase, Web of Science, MEDLINE, and China National Knowledge Infrastructure (CNKI), were searched to identify relevant articles from inception to October 2021. The search was limited to human studies published in English and included using the following terms: *“*recurrent pregnancy loss*”*, *“*repeated pregnancy loss*”*, *“*recurrent abortions*”*, *“*recurrent spontaneous abortion*”*, *“*recurrent miscarriage*”*, *“*sperm DNA fragmentation*”*, *“*sperm DNA integrity*”*, *“*sperm DNA damage*”*, *“*SDF*”*, *“*DFI*”*, *“*traditional semen parameters*”*, and *“*conventional semen parameters*”*.　

### Selection Criteria

Studies that met the following criteria were included in this study: (1) original research; (2) the topic is unexplained RM; (3) natural conception; and (4) the data for traditional semen parameters and SDF are expressed as the means with standard deviations (SDs). The exclusion criteria were as follows: (1) reviews, letters, editorials, and abstracts; (2) inaccessible full articles; (3) case-only studies; and (4) duplicate publications.

### Selection of Publications

Based on the predefined inclusion/exclusion criteria, all publications were independently selected for eligibility by two authors (Y.D. and J.L.). After removing duplicates, articles were selected by reviewing the titles and abstracts. The remaining publications were retrieved for full-text assessment if their appropriateness could not be determined. Any discrepancy was resolved through discussion with the third reviewer (E.Y.).

### Data Extraction

From the eligible publications, data were extracted independently by two authors (Y.D. and J.L.). Any discrepancy between the two authors (Y.D. and J.L.) was resolved by discussion with the third reviewer (E.Y.). The following information was collected for each eligible publication: name of the first author, publication year, country of origin, ethnicity group, type of study design, sample size, and methods used to evaluate SDF. The main characteristics of the included studies are listed in **Table 1**.

**Table 1:** Main characteristics of the included studies in the meta-analysis.

Author (year)	Country	Ethnicity	Study design	Cases	Controls	Sample size Cases/controls	Samples for DFI	Assay	Quality score
Absalan et al. (19)	Iran	Asian	Prospective	RPL≥ 3 times	Fertile	30/30	Fresh semen	SCD	7
Bareh et al. (24)	USA	Caucasian	Prospective	RPL≥ 2times	≥1 live birth	26/31	Fresh semen	TUNEL	7
Bhattacharya et al. (29)	India	Asian	Prospective	RPL≥ 2times	≥1 live birth	74/65	Fresh semen	AOT	7
Brahem et al. (25)	Tunisia	African	Prospective	RPL≥ 2times	Fertile	31/20	Frozen semen	TUNEL	7
Carlini et al. (26)	Italy	Caucasian	Prospective	RPL≥ 2times	≥1 live birth	112/114	Fresh semen	TUNEL	8
Carrell et al. (38)	USA	Caucasian	Prospective	RPL≥ 3 times	≥1 live birth	21/26	Frozen semen	TUNEL	7
Coughlan et al. (20)	UK	Caucasian	Prospective	RPL≥ 3 times	≥1 live birth	16/7	Fresh semen	SCD	8
Eisenberg et al. (30)	USA	Caucasian	Prospective	RPL≥ 2times	Currently pregnant	14/246	Frozen semen	SCSA	9
Gil-villa et al. (31)	USA	Caucasian	Prospective	RPL≥ 2times	≥1 live birth	23/11	Frozen semen	SCSA	7
Imam et al. (32)	India	Asian	Retrospective	RPL≥ 3 times	≥1 live birth	20/20	Frozen semen	SCSA	8
Kamkar et al. (27)	Iran	Asian	Retrospective	RPL≥ 2times	≥1 live birth	42/42	Frozen semen	SCSA and TUNEL	7
Khadem et al. (21)	Iran	Asian	Prospective	RPL≥ 3 times	Currently pregnant	30/30	Fresh semen	SCD	8
Kumar et al. (33)	India	Asian	Prospective	RPL≥ 3 times	≥1 live birth	45/20	Frozen semen	SCSA	7
Ribas-Maynou et al. (22)	Spain	Caucasian	Prospective	RPL≥ 2times	≥1 live birth	20/25	Frozen semen	SCD	8
Ruixue et al. (36)	China	Asian	Prospective	RPL≥ 3 times	Currently pregnant	68/63	Fresh semen	AB staining	7
Venkatesh et al. (34)	India	Asian	Prospective	RPL≥ 3times	≥1 live birth	16/20	Frozen semen	SCSA	7
Zhang et al. (23)	China	Asian	Prospective	RPL≥ 2times	≥1 live birth	111/30	Fresh semen	SCD	7
Zhu et al. (35)	China	Asian	Retrospective	RPL≥ 2times	Fertile	461/411	Fresh semen	SCSA	8
Zidi-Jrah et al. (28)	Tunisia	African	Prospective	RPL≥ 2times	≥1 live birth	22/20	Frozen semen	TUNEL	7

SCSA, sperm chromatin structure assay; SCD, sperm chromatin dispersion; TUNEL, terminal TdT-mediated dUTP-nick-end labeling; AOT, acridine orange test; AB staining, aniline blue staining.

### Quality Assessment of the Included Publications

Quality assessments were performed using the Newcastle–Ottawa Scale (NOS) (39). A NOS score of ≥6 was considered high quality (40).

### Statistical Analysis

All analyses were performed using Stata/SE 12.0 (StataCorp, College Station, Texas, USA). The heterogeneity between publications was calculated using the I^2^ statistic and Cochran’s Q test. Heterogeneity was considered significant at *P*<0.10 and/or *I^2^* <50%. Based on the heterogeneity assessment, random- or fixed-effects models were selected to calculate the weighted mean differences (WMDs) and their corresponding 95% confidence intervals (CIs). To explore the potential sources of heterogeneity, subgroup analyses were performed. To estimate the stability of the pooled results, a sensitivity analysis was conducted by excluding each publication. To estimate the possible publication bias, Egger’s regression test and Begg’s funnel plot were used. Statistical significance was set at *P*<0.05.

## Results

### Selection of Publications

**Figure 1:**
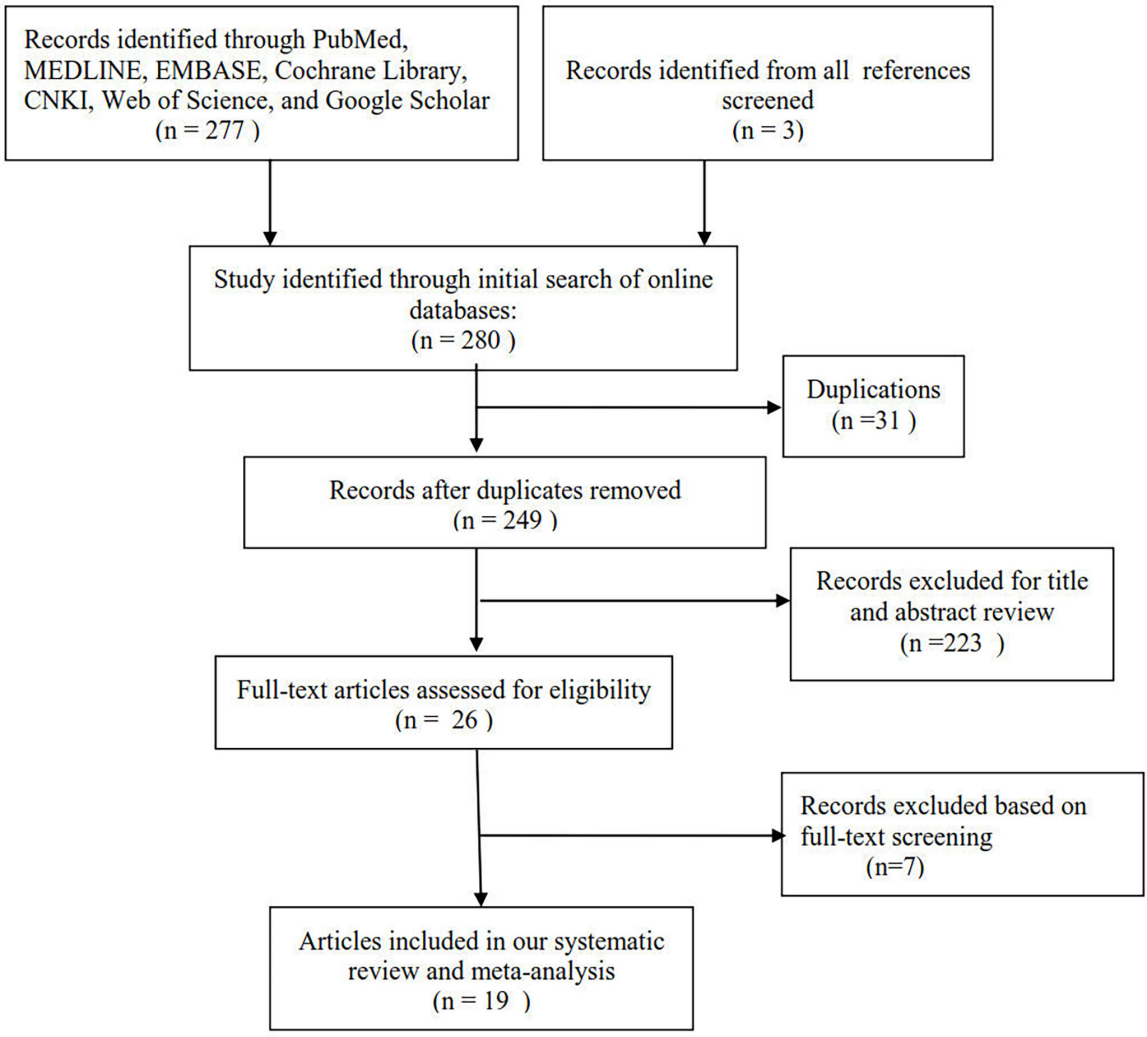
Selection process for eligible publications.

**Figure 1** shows the selection process of eligible publications. Based on our search strategy, 280 publications were initially identified through a database search. A total of 249 titles and abstracts of publications were reviewed after removing 31 duplicates. After screening the titles and abstracts of publications, 26 potentially relevant publications were found. The remaining publications were retrieved for full-text assessment. After full-text assessment of the remaining publications, 7 publications were excluded for various reasons. A total of 19 publications were finally included in the meta-analysis, which involved 2413 subjects (1182 couples with unexplained RM and 1231 couples without RM).

### Characteristics of the Eligible Publications

**Table 1** presents the main characteristics of the eligible publications. All included articles were of relatively high quality. None of the couples had received assisted reproductive treatments. Male partners of couples without unexplained RM had proven natural fertility with one or more live births. Female partners were under 40 years of age in all eligible studies. Female partners had normal ovarian function and a normal uterus (demonstrated by hysteroscopy, hysterosalpingography, and/or hysterosonography). Female partners with any of the following were excluded: abnormal karyotypes and uterine structural abnormalities.

### Relation Between Traditional Semen Parameters and Unexplained RM

**Figure 2:**
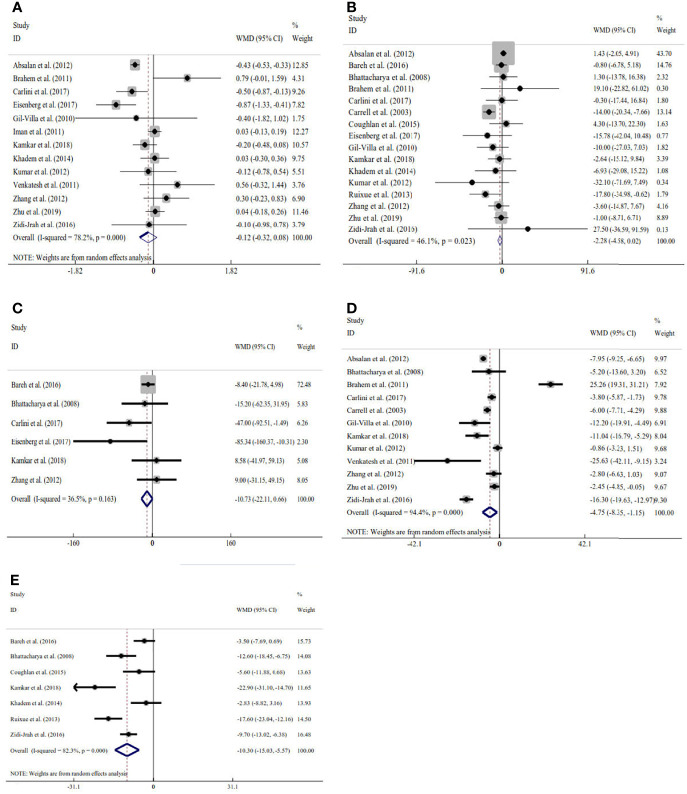
Meta-analysis of the relations between traditional semen parameters and unexplained recurrent miscarriage. **(A)** Relation between volume and unexplained recurrent miscarriage; **(B)** relation between sperm concentration and unexplained recurrent miscarriage; **(C)** relation between total sperm count and unexplained recurrent miscarriage; **(D)** relation between progressive motility and unexplained recurrent miscarriage; and **(E)** relation between total motility and unexplained recurrent miscarriage.

**Figure 3:**
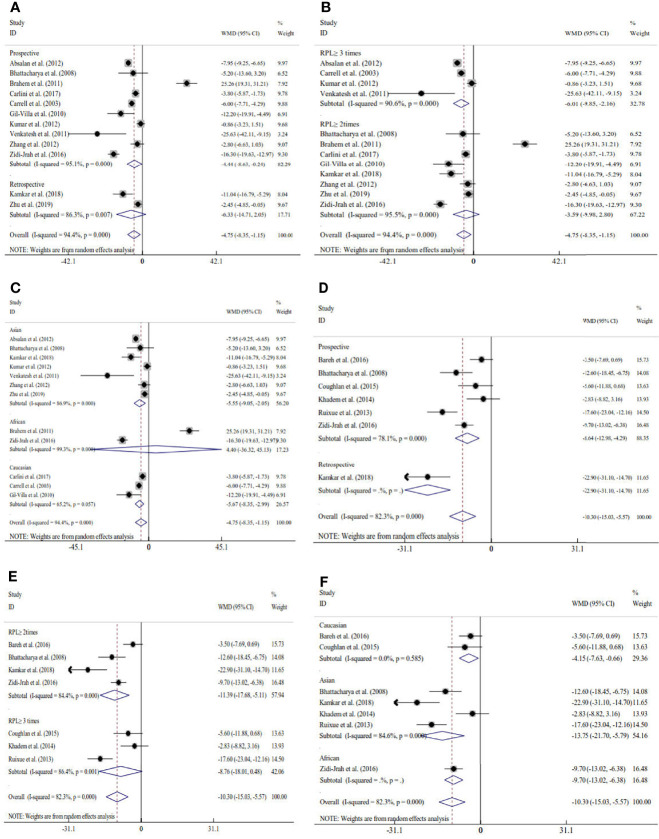
Subgroup analyses for progressive motility by the type of study design **(A)**, definition of recurrent miscarriage **(B)**, and ethnicity **(C)**; subgroup analyses for total motility by type of study design **(D)**, definition of recurrent miscarriage **(E)**, and ethnicity **(F)**.

Sixteen studies explored the relation between traditional semen parameters and unexplained RM. For all eligible studies, the evaluation of traditional semen parameters was performed in fresh semen samples. The pooled results showed that there were no relations between unexplained RM and semen volume (WMD=-0.12, 95% CI=-0.32 to 0.08, *P*>0.05), sperm concentration (WMD=-2.28, 95% CI=-4.58 to 0.02, *P>*0.05), and total sperm count (WMD=-10.73, 95% CI=-22.11 to 0.66, *P*>0.05) (**Figure 2**). However, the pooled results showed that there were significant relations between unexplained RM and progressive motility (WMD=-4.75, 95% CI=-8.35 to -1.15, *P*<0.05) and total motility (WMD=-10.30, 95% CI=-15.03 to -5.57, *P*<0.05) (**Figure 2**). Since significant heterogeneity was observed for the total and progressive motility (*I^2^ = *82.3%, *P*<0.001; *I^2^ = *99.4%, *P*<0.001), subgroup analyses were performed by the study design type, RM definition, and ethnicity to explore the source of heterogeneity (**Figure 2**). For the majority of the subgroups, the percentages of total and progressive motility were significantly lower in couples with unexplained RM than in couples without RM (**Figures 3A–F**).

### Relation Between SDF and Unexplained RM

**Figure 4:**
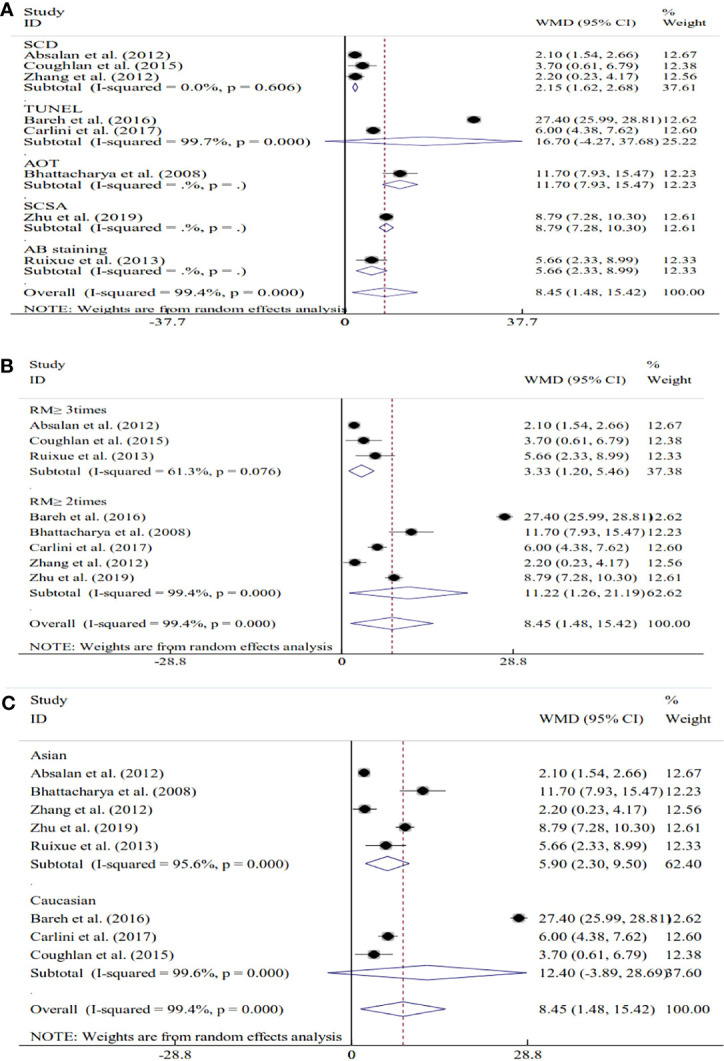
Subgroup analyses based on the sperm DNA fragmentation assay **(A)**, the definition of recurrent miscarriage **(B)**, and ethnicity **(C)**.

Seventeen studies explored the relations between SDF and unexplained RM. For 8 of these studies, SDF was assessed using fresh semen samples. The pooled results showed that couples with unexplained RM had significantly increased levels of SDF compared with couples without RM (WMD=8.45, 95% CI=1.48 to 15.42, *P*=0.018) (**Table 2** and **Figure 4**). Because significant heterogeneity was observed for SDF (*I^2^ = *99.4%, *P*<0.001), subgroup analyses were performed by the assay type, RM definition, and ethnicity to explore the source of heterogeneity (**Table 2** and **Figure 4**). Subgroup analysis by SDF assay also showed a significant association between couples with and without RM for the SCD assay (WMD=2.15, 95% CI=1.62 to 2.68, *P*<0.001) (**Table 2** and **Figure 4A**). The subgroup analysis by the definition of RM showed that couples with a history of RM ≥2 times and ≥3 times had significantly increased levels of SDF compared with couples without RM (WMD=11.22, 95% CI=1.26 to 21.19, *P*=0.027 and WMD=3.33, 95% CI=1.20 to 5.46, *P*=0.002) (**Table 2** and **Figure 4B**). The subgroup analysis by ethnicity also showed similar results to the overall analysis in the Asian subgroup (WMD=5.90, 95% CI=2.30 to 9.50, *P*=0.001) (**Table 2** and **Figure 4C**).

**Table 2:** Subgroup analyses by sperm DNA fragmentation assay, definition of recurrent miscarriage, sperm preservation and ethnicity.

Outcomes	*N*	Model used	Heterogeneity		Pooled WMD		Begg’s test *P*
			I^2^ (%)	*P* value	WMD (95 CI)	*P* value	
Assay							
SCD	3	Fixed-effects	0.0	0.606	2.15 (1.62 to 2.68)	<0.001	
TUNEL	2	Random-effects	99.7	<0.001	16.70 (-4.27 to 37.68)	0.118	
AOT	1	NA	NA	NA	NA	NA	
SCSA	1	NA	NA	NA	NA	NA	
AB staining	1	NA	NA	NA	NA	NA	
Overall	8	Random-effects	99.4	<0.001	8.45 (1.48 to 15.42)	0.018	1.000
Definition of RPL							
RPL≥3 times	3	Random-effects	61.3	0.0076	3.33 (1.20 to 5.46)	0.002	
RPL≥2 times	5	Random-effects	99.4	<0.001	11.22 (1.26 to 21.19)	0.027	
Overall	8	Random-effects	99.4	<0.001	8.45 (1.48 to 15.42)	0.018	1,000
Ethnicity							
Asian	5	Random-effects	95.6	<0.001	5.90 (2.30 to 9.50)	0.001	
Caucasian	3	Random-effects	99.6	<0.001	12.40 (-3.89 to 28.69)	0.136	
Overall	8	Random-effects	99.4	<0.001	8.45 (1.48 to 15.42)	0.018	1.000

### Sensitivity Analyses

**Figure 5:**
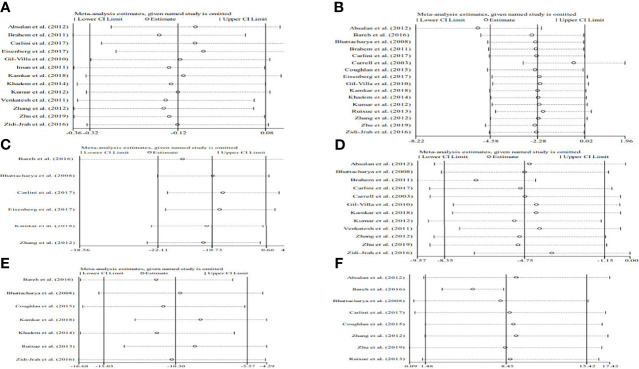
Begg’s funnel plot of the relationship among volume **(A)**, sperm concentration **(B)**, total sperm count **(C)**, progressive motility **(D)**, total motility **(E)**, and sperm DNA fragmentation **(F)** and unexplained recurrent miscarriage.

The sensitivity analysis showed that the pooled results were stable and reliable (**Figure 5**).

### Publication Bias

**Figure 6:**
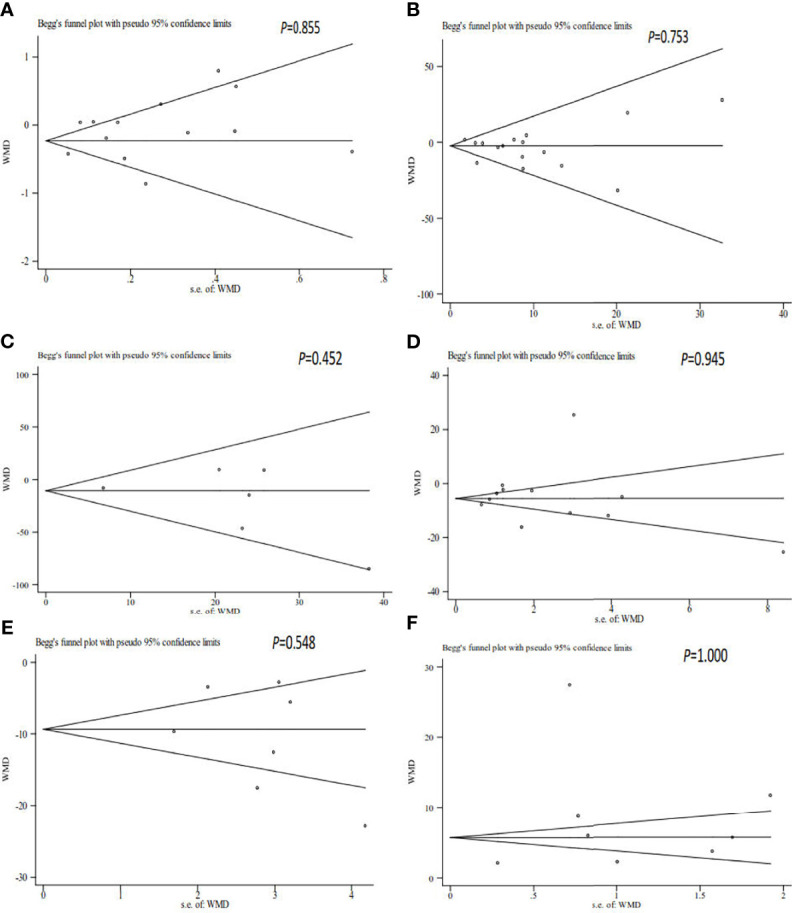
Sensitivity analysis of the relationship among volume **(A)**, sperm concentration **(B)**, total sperm count **(C)**, progressive motility **(D)**, total motility **(E)**, and sperm DNA fragmentation **(F)** and unexplained recurrent miscarriage.

As shown in **Table 2** and **Figure 6**, our results showed that there was no publication bias for the semen volume, sperm concentration, total sperm count, progressive motility, total motility, and SDF.

## Discussion

RM affects approximately 1% of couples trying to conceive (5). In almost half of the cases of RM, the etiology of the affected couples remains unclear (1). The role of female factors in RM has been studied intensively, but the role of male factors has been less thoroughly investigated (6–8).

Some studies have reported that male partners of couples with unexplained RM had significantly decreased levels of semen volume (26) and progressive motility (19, 27, 31, 34, 35) compared with couples without RM, but significant differences were not observed in sperm concentration (19–21, 23–31, 33, 35, 36, 38), total sperm count (23, 24, 26, 27, 29, 30) and total motility (20, 21, 24, 36) between the two groups. Some studies reported that couples with unexplained RM had significantly increased levels of sperm concentration (34) and total motility (25, 27–29) compared with couples without RM, but significant differences were not observed in semen volume (21, 23, 25, 27, 28, 30–35) and progressive motility (23, 26, 29) between the two groups. The combined results of this meta-analysis showed that couples with unexplained RM had significantly decreased levels of progressive motility and total motility than those of couples without RM. The combined results demonstrate that women whose partners had a higher percentage of progressive motility and total motility were more likely to have a successful pregnancy while women whose partners had a lower percentage of progressive motility and total motility were less likely to conceive and/or more likely to experience pregnancy loss.

Marked between-study heterogeneity was observed for progressive motility and total motility, and it could not be ignored. Therefore, subgroup analyses by the study design type, RM definition, and ethnicity were performed to explore the source of heterogeneity. However, heterogeneity was still observed despite performing the subgroup analyses. Such heterogeneity may be explained by differences in age and number of participants, duration of sexual abstinence, ethnicity, lifestyle habits, laboratory techniques, etc.

However, approximately 15% of male factor infertility patients show normal parameters in their ejaculates (41), suggesting that conventional semen parameters are poor predictors of reproductive outcome and that a definitive diagnosis of male infertility cannot be made by a routine semen analysis alone, which is because several factors other than conventional semen parameters affect the fertilization ability of spermatozoa.

Routine semen analysis does not assess all aspects of sperm quality. SDF is used to assess the integrity of sperm chromatin and may be a better predictor of male fertility and reproductive outcomes than conventional semen parameters. Sperm DNA integrity plays an important role in the initiation and maintenance of pregnancy (42). The study of sperm DNA integrity may be important for understanding the pathogenesis of unexplained RM. However, the relationship between sperm DNA integrity and unexplained RM remains controversial. Some studies (19, 20, 22, 24–29, 32, 33, 35, 36, 38) have reported that couples with unexplained RM had significantly increased levels of SDF compared with those of couples without RM. However, other studies (23, 30, 31) have reported no significant differences in SDF between couples with and without RM. For these studies, SDF was assessed using fresh or cryopreserved semen samples. The cryopreservation process can alter the sperm quality, particularly the motility and sperm DNA integrity (43–45). Only those studies that assessed SDF with fresh semen samples were included in this meta-analysis to evaluate the relationship between SDF and unexplained RM. The combined results of this meta-analysis demonstrated that couples with unexplained RM had significantly increased levels of SDF compared with couples without RM. Our results demonstrated that women whose partners had a lower percentage of SDF were more likely to have a successful pregnancy while women whose partners had a higher percentage of SDF were more likely to experience pregnancy loss. Our results also suggested that male factors may be involved in the pathogenesis of RM and that SDF might be used as a tool to evaluate the risk of RM. However, future large prospective studies are needed to evaluate the impact of elevated SDF on the risk of RM.

Marked between-study heterogeneity was observed, and it could not be ignored. Several factors may account for the measured heterogeneity. First, there are several methods used to assess SDF. Second, there are two definitions of unexplained RM. Third, the subjects included in the studies were of diverse ethnic backgrounds. All of these factors may have significantly affected the between-study heterogeneity. Subgroup analyses by the assay type, RM definition, and ethnicity were performed to explore the source of heterogeneity. However, heterogeneity was still observed despite performing these subgroup analyses. The results of the subgroups by the definition of RM showed that the couples with a history of RM≥2 times and ≥3 times had significantly increased levels of SDF. Given the limited sample size of the included studies and the significant heterogeneity between studies, further large prospective cohort studies are needed to validate these findings.

There were four strengths of this meta-analysis. First, more reliable results can be obtained as a result of the large sample size. Second, we also assessed the relation between traditional semen parameters and unexplained RM. Third, the subgroup analyses by the assay type, RM definition, and ethnicity were also conducted in this study. Fourth, no publication bias was found in this meta-analysis.

This meta-analysis has two limitations. First, between-study heterogeneity was found despite using strict inclusion/exclusion criteria. Second, the number of included publications was small in some subgroups.

Couples with unexplained RM had significantly increased levels of SDF compared with couples without RM, and they also had significantly decreased progressive motility and total motility. The SDF assay may be considered for inclusion in evaluations of couples with unexplained RM. Future large prospective studies are needed to evaluate the impact of elevated SDF on the risk of RM.

## Data Availability Statement

The original contributions presented in the study are included in the article/supplementary material. Further inquiries can be directed to the corresponding authors.

## Author Contributions

All authors contributed to the study’s conception and design. YD and JL participated in drafting the manuscript and the study design. EY and YL searched and selected the relevant articles. YS and LZ contributed to the statistical analysis. All authors contributed to the article and approved the submitted version.

## Funding

By Joint Construction Project of Henan Medical Science and Technology Research Plan (LHGJ20190396).

## Conflict of Interest

The authors declare that the research was conducted in the absence of any commercial or financial relationships that could be construed as a potential conflict of interest.

## Publisher’s Note

All claims expressed in this article are solely those of the authors and do not necessarily represent those of their affiliated organizations, or those of the publisher, the editors and the reviewers. Any product that may be evaluated in this article, or claim that may be made by its manufacturer, is not guaranteed or endorsed by the publisher.
